# Supplementary material for: The Theoretical Framework of the Clinical Pilates Exercise Method in Managing Non-Specific Chronic Low Back Pain: A Narrative Review
Source: Biology (Basel). 2021 Oct 25;10(11):1096. doi: 10.3390/biology10111096 (PMC8615180; doi:10.3390/biology10111096)
Supplement: Supplementary file 1 [file biology-10-01096-s001.zip › biology-1420865-supplementary-revised 10.26/Supp files/Supplementary file 1 List of non Clinical Pilates studies search yield.pdf]

Supplementary file 1: List of non-Clinical Pilates studies search yield.

PubMed search (9 studies)

1. Owen PJ, Miller CT, Mundell NL, Verswijveren SJJM, Tagliaferri SD, Brisby H, Bowe SJ, Belavy DL. Which specific modes of exercise training are most effective for treating low back pain? Network meta-analysis. Br J Sports Med. 2020 Nov;54(21):1279-1287. doi: 10.1136/bjsports-2019-100886. Epub 2019 Oct 30. PMID: 31666220; PMCID: PMC7588406.

2. Yamato TP, Maher CG, Saragiotto BT, Hancock MJ, Ostelo RW, Cabral CM, Menezes Costa LC, Costa LO. Pilates for low back pain. Cochrane Database Syst Rev. 2015 Jul 2;2015(7):CD010265. doi: 10.1002/14651858.CD010265.pub2. PMID: 26133923; PMCID: PMC8078578.

3. Mostagi FQ, Dias JM, Pereira LM, Obara K, Mazuquin BF, Silva MF, Silva MA, de Campos RR, Barreto MS, Nogueira JF, Lima TB, Carregaro RL, Cardoso JR. Pilates versus general exercise effectiveness on pain and functionality in non-specific chronic low back pain subjects. J Bodyw Mov Ther. 2015 Oct;19(4):636-45. doi:10.1016/j.jbmt.2014.11.009. Epub 2014 Nov 18. PMID: 26592221.

4. Yamato TP, Maher CG, Saragiotto BT, Hancock MJ, Ostelo RW, Cabral CM, Costa LC, Costa LO. Pilates for low back pain. Sao Paulo Med J. 2016 Jul- Aug;134(4):366-7. doi: 10.1590/1516-3180.20161344T1. PMID: 27557145.

5. Mazloum V, Sahebozamani M, Barati A, Nakhaee N, Rabiei P. The effects of selective Pilates versus extension-based exercises on rehabilitation of low back pain. J Bodyw Mov Ther. 2018 Oct;22(4):999-1003. doi:10.1016/j.jbmt.2017.09.012. Epub 2017 Sep 20. PMID: 30368347.

6. Miyamoto GC, Franco KFM, van Dongen JM, Franco YRDS, de Oliveira NTB, Amaral DDV, Branco ANC, da Silva ML, van Tulder MW, Cabral CMN. Different doses of Pilates-based exercise therapy for chronic low back pain: a randomised controlled trial with economic evaluation. Br J Sports Med. 2018 Jul;52(13):859-868. doi: 10.1136/bjsports-2017-098825. Epub 2018 Mar 10. PMID: 29525763.

7. La Touche R, Escalante K, Linares MT. Treating non-specific chronic low back pain through the Pilates Method. J Bodyw Mov Ther. 2008 Oct;12(4):364-70. doi:10.1016/j.jbmt.2007.11.004. Epub 2008 Feb 1. PMID: 19083695.

8. Baillie L, Bacon CJ, Hewitt CM, Moran RW. Predictors of functional improvement in people with chronic low back pain following a graded Pilates-based exercise programme. J Bodyw Mov Ther. 2019 Jan;23(1):211-218. doi:10.1016/j.jbmt.2018.06.007. Epub 2018 Jun 30. PMID: 30691755.

9. Donzelli S, Di Domenica E, Cova AM, Galletti R, Giunta N. Two different techniques in the rehabilitation treatment of low back pain: a randomized controlled trial. Eura Medicophys. 2006 Sep;42(3):205-10. PMID: 17039216.

Supplementary file 1: List of non-Clinical Pilates studies search yield.

Cochrane search (13 studies)

Reviews:

1. Geneen LJ, Moore RA, Clarke C, Martin D, Colvin LA, Smith BH. Physical activity and exercise for chronic pain in adults: an overview of Cochrane Reviews. Cochrane Database of Systematic Reviews. 2017(4).

2. Macedo LG, Saragiotto BT, Yamato TP, Costa LOP, Menezes Costa LC, Ostelo R, et al. Motor control exercise for acute non-specific low back pain. Cochrane Database of Systematic Reviews. 2016(2).

3. Saragiotto BT, Maher CG, Yamato TP, Costa LOP, Menezes Costa LC, Ostelo R, et al. Motor control exercise for chronic non-specific low-back pain. Cochrane Database of Systematic Reviews. 2016(1).

4. Wieland LS, Skoetz N, Pilkington K, Vempati R, D'Adamo CR, Berman BM. Yoga treatment for chronic non-specific low back pain. Cochrane Database of Systematic Reviews. 2017(1).

5. Yamato TP, Maher CG, Saragiotto BT, Hancock MJ, Ostelo R, Cabral CMN, et al. Pilates for low back pain. Cochrane Database of Systematic Reviews. 2015(7).

Trials:

1. Miranda IF, Souza C, Schneider AT, Chagas LC, Loss JF. Comparison of low back mobility and stability exercises from Pilates in non-specific low back pain: A study protocol of a randomized controlled trial. Complement Ther Clin Pract. 2018;31:360-8.

2. Valenza MC, Rodriguez-Torres J, Cabrera-Martos I, Diaz-Pelegrina A, Aguilar-Ferrandiz ME, Castellote-Caballero Y. Results of a Pilates exercise program in patients with chronic non-specific low back pain: a randomized controlled trial. Clin Rehabil. 2017;31(6):753-60.

3. Donzelli S, Di Domenica E, Cova AM, Galletti R, Giunta N. Two different techniques in the rehabilitation treatment of low back pain: a randomized controlled trial. Eura Medicophys. 2006;42(3):205-10.

4. Shahrjerdi S, Golpayegani M, Daghaghzadeh A, Karami A. The effect of Pilates-based exercises on pain, functioning and lumbar lordosis in women with non-specific chronic low back pain and hyperlordosis. J Adv Med Biomed Res. 2014;22(94):120-31.

5. Tottoli CR, van Tulder M, Silva ENd, Marques YA, Martins WR, Luiz Carregaro R. Effectiveness and cost-effectiveness of Pilates versus home-based exercises in individuals with chronic non-specific low back pain: randomised controlled trial protocol. European Journal of Physiotherapy. 2021;23(2):95-101.

6. Miyamoto GC, Franco KFM, van Dongen JM, Franco Y, de Oliveira NTB, Amaral DDV, et al. Different doses of Pilates-based exercise therapy for chronic low back pain: a randomised controlled trial with economic evaluation. Br J Sports Med. 2018;52(13):859-68.

7. Batibay S, Kulcu DG, Kaleoglu O, Mesci N. Effect of Pilates mat exercise and home exercise programs on pain, functional level, and core muscle thickness in women with chronic low back pain. J Orthop Sci. 2020.

8. Mostagi FQ, Dias JM, Pereira LM, Obara K, Mazuquin BF, Silva MF, et al. Pilates versus general exercise effectiveness on pain and functionality in non-specific chronic low back pain subjects. J Bodyw Mov Ther. 2015;19(4):636-45.

9. Patti A, Bianco A, Paoli A, Messina G, Montalto MA, Bellafiore M, et al. Pain Perception and Stabilometric Parameters in People With Chronic Low Back Pain After a Pilates Exercise Program: A Randomized Controlled Trial. Medicine (Baltimore). 2016;95(2):e2414.

Supplementary file 1: List of non-Clinical Pilates studies search yield.

10. Mazloum V, Sahebozamani M, Barati A, Nakhaee N, Rabiei P. The effects of selective Pilates versus extension-based exercises on rehabilitation of low back pain. J Bodyw Mov Ther. 2018;22(4):999-1003.

*Conference proceedings, trial registration studies and study duplicates were excluded.
