# Supplementary material for: The Theoretical Framework of the Clinical Pilates Exercise Method in Managing Non-Specific Chronic Low Back Pain: A Narrative Review
Source: Biology (Basel). 2021 Oct 25;10(11):1096. doi: 10.3390/biology10111096 (PMC8615180; doi:10.3390/biology10111096)
Supplement: Supplementary file 1 [file biology-10-01096-s001.zip › biology-1420865-supplementary-revised 10.26/Supp files/Supplementary file 2 Included studies tables.pdf]

Supplementary file 2: Appraisal and study summary of included studies.

Table S1: Appraisal of Clinical Pilates studies with PEDro scale.

S/N	Author/Year	Q1*	Q2	Q3	Q4	Q5	Q6	Q7	Q8	Q9	Q10	Q11	Total, /10
1	Taylor et al. 2011	Yes	No	No	No	No	No	No	Yes	Yes	No	Yes	3
2	Wajswelner et al. 2012	Yes	Yes	Yes	Yes	No	No	No	Yes	Yes	Yes	Yes	7
3	Devasahayam et al. 2016	Yes	Yes	Yes	Yes	No	Yes	No	No	No	Yes	Yes	6

*Not included in total score.

Supplementary file 2: Appraisal and study summary of included studies.

Table S2: Study characteristics of Clinical Pilates studies included in the review.

S/N	Author / Year	Study population / design	Intervention settings	Number of intervention exercises	Number of home exercises	Exercise principles (frequency, intensity and duration)	Outcome time-points (from baseline)	Outcome measures used	Intervention efficacy summary	Level of evidence[#]
1	Taylor et al. 2011	15 subjects; mean age (SD) is 39.8(10.9) years, 8 females; single-arm study.	Clinical outpatient / New Zealand / 1-to-1 session.	Not stated	0	- 2x/week for 6 weeks;<br>- Moderate intensity (strength or endurance exercise principle);<br>- 1 hour.	1.5, 3 and 9 months	ODI, pain VAS and quality of life.	ODI, pain VAS and quality of life (all physical health components and 2 mental health components - vitality and mental health) improved. Longer-term carry-over effect was observed.	4
2	Wajswelner et al. 2012	87 subjects; mean age (SD): 48.9(16.4) years for general exercise group and 49.3(14.1) years for intervention group, 48 females; RCT.	Clinical outpatient / Australia / Group of 4 subjects (first session 1-to-1).	6 to 12	1 to 4 mat exercises	- 2x/week for 6 weeks;<br>- Intensity not stated;<br>- 1 hour.	1.5, 3 and 6 months	Quebec scale, pain NRS, PSFS, pain self-efficacy scale and quality of life.	No between-group differences. Both groups improved from baseline. Longer-term carry-over effect was observed.	1b
3	Devasahayam et al. 2016	21 subjects (6 were not analysed); median age (IQR): 61.5(46 to 69.5) years for general exercise (control) group and 48(39 to 62.5) for intervention group, gender not specified; RCT.	Clinical outpatient / Singapore / 1-to-1 session.	Not stated	0	- 1x/week for 6 weeks;<br>- Intensity not stated;<br>- 0.5 hour.	1.5 month	Pain NRS, global perceived effect scale, PSFS and WOMAC.	No between-group differences. Statistical significant improvements from baseline were found for pain NRS in the control group, WOMAC for the Pilates group, and global perceived effect scale and PSFS for both groups.	2b

[#]Classified based on Oxford Centre for Evidence-Based Medicine: Level of Evidence (March 2009).

ODI: Oswetry Disability Index; VAS: Visual analogue scale; NRS: Numeric rating scale; PSFS: Patient Specific Functional Scale; WOMAC: Western Ontario and McMaster Universities Osteoarthritis Index.
