# Supplementary material for: The Theoretical Framework of the Clinical Pilates Exercise Method in Managing Non-Specific Chronic Low Back Pain: A Narrative Review
Source: Biology (Basel). 2021 Oct 25;10(11):1096. doi: 10.3390/biology10111096 (PMC8615180; doi:10.3390/biology10111096)

Supplementary file 3: Directional preference assessment algorithms.

Figure S1: Transverse axis directional preference assessment algorithm.

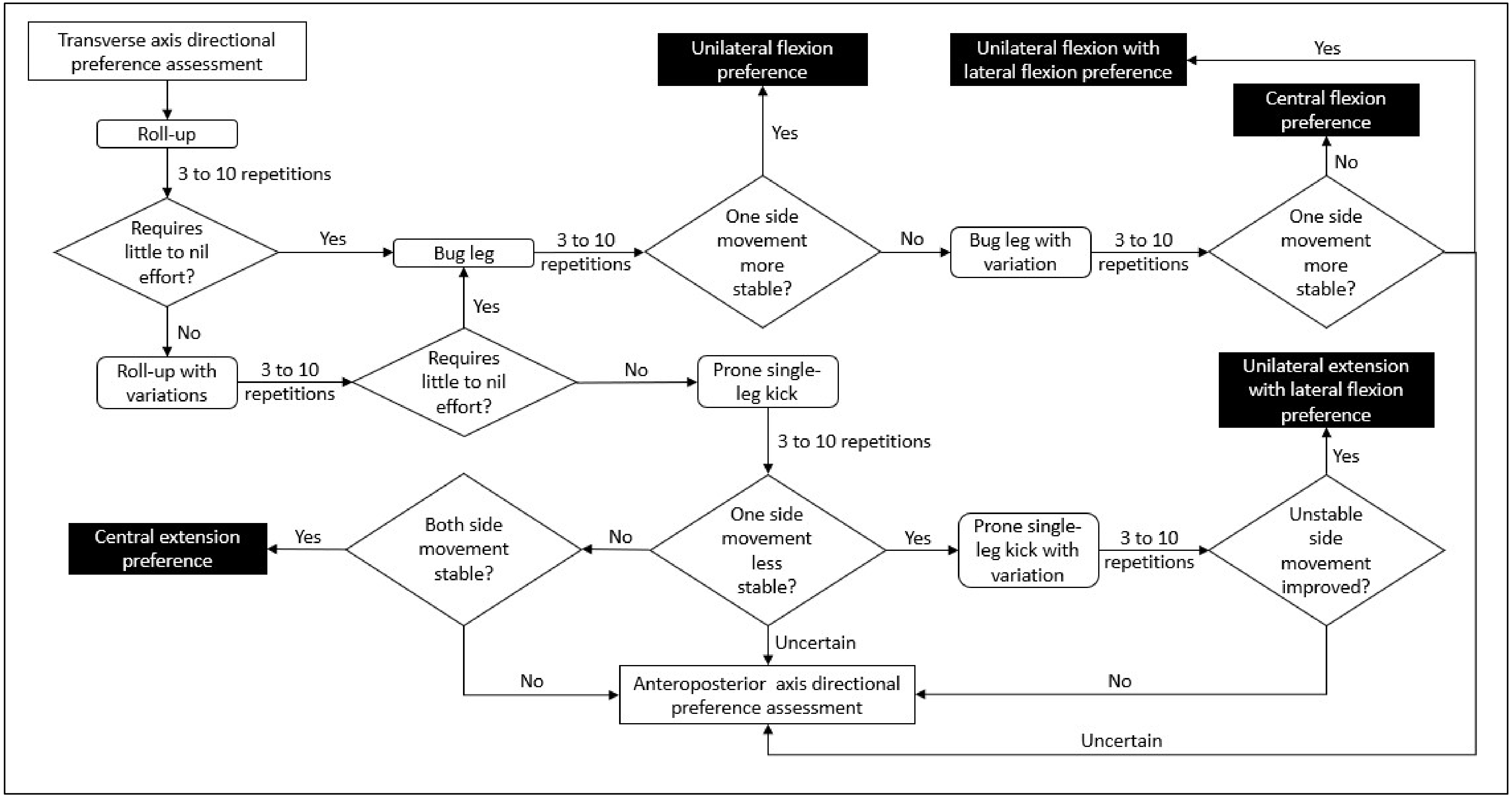

Supplementary file 3: Directional preference assessment algorithms.

Figure S2: Anteroposterior axis directional preference assessment algorithm.

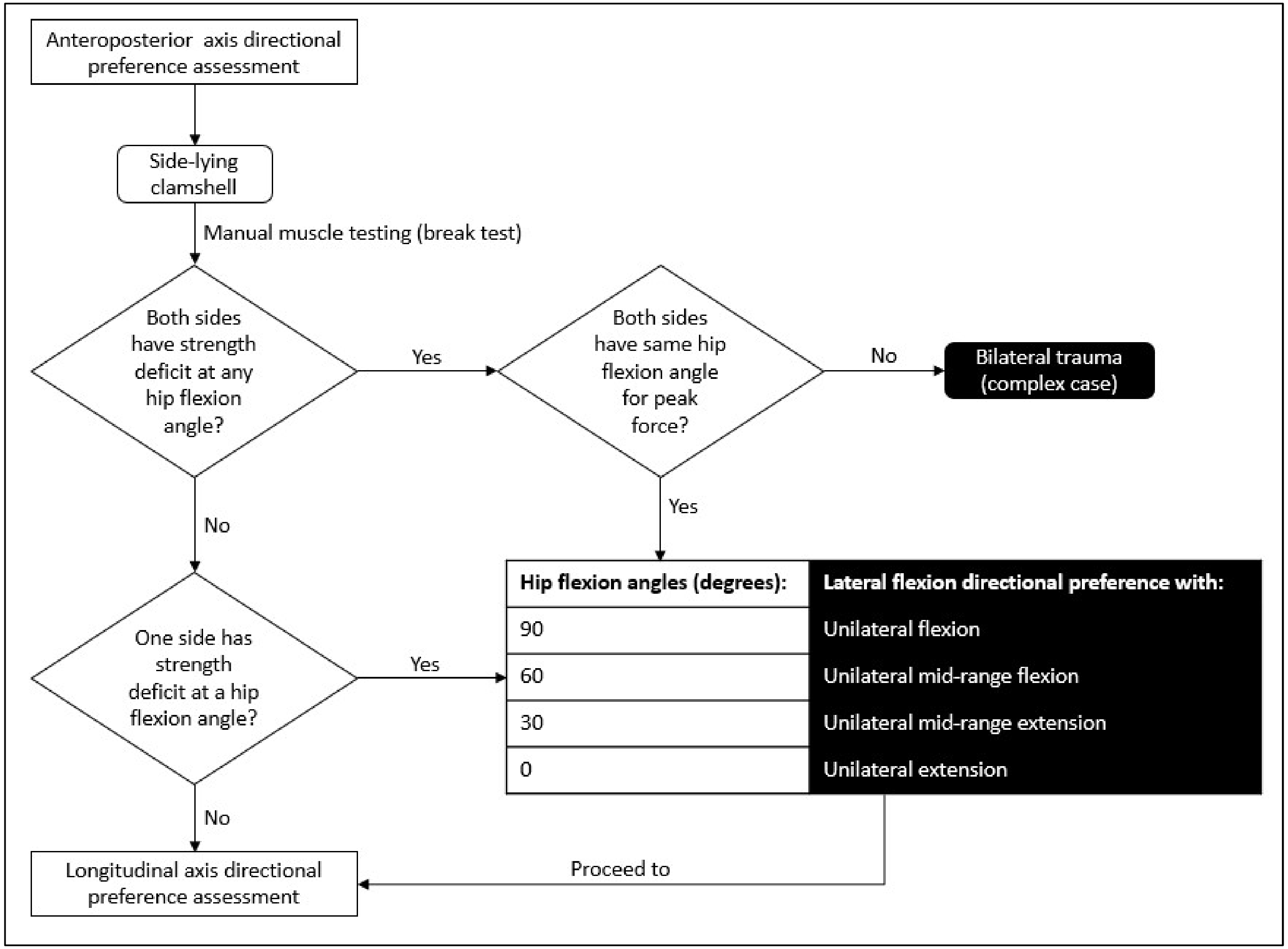

Supplementary file 3: Directional preference assessment algorithms.

<u>Figure S3: Longitudinal axis directional preference assessment algorithm.</u>

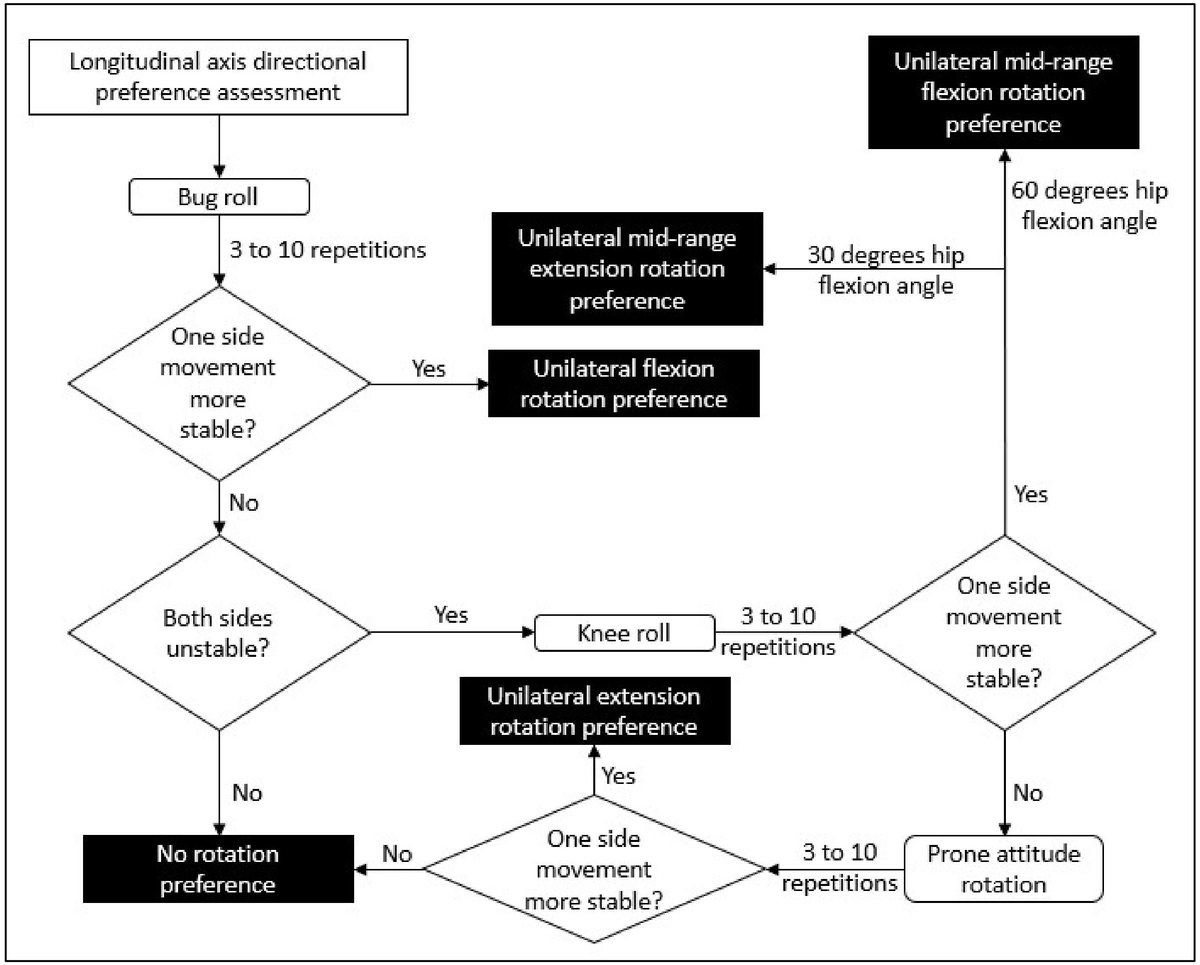

Supplement: Supplementary file 1 [file biology-10-01096-s001.zip › biology-1420865-supplementary-revised 10.26/Supp files/Supplementary file 3 Figure S1-3 Directional preference Ax algorithm.pdf]
